# Editorial

**Published:** 1916-08

**Authors:** 


### EDITORIAL

The Atlanta Journal-Record of Medicine has its Business Office at 23 West Alabama Street where all business communications should be sent.

Remittances are payable to The Journal-Record of Medicine.

Concerning Original Communications and Editorial matters, address Richard R. Daly, M. D., 708 Empire Life Building, Atlanta, Ga.

Reprints of Original Articles will be furnished at cost price. Requests for them had best be made on the Manuscript.

Upon request, we will present, postpaid, to each contributor of an original article, twenty copies of The Journal-Record of Medicine containing such article.

### JOHN B. MURPHY.

At the age of fifty-eight, Dr. John B. Murphy died August the 11, 1916. In his death the greatest surgeon in America passed away, not however, without leaving lasting landmarks in his well chosen profession. He was graduated from the Rush Medical College in 1879, and after serving as resident interne in the Cook County Hospital, Chicago, he went to Vienna, where he studied under the master German surgeon Billroth from whose charming personality and original mind he seemed to have absorbed these characteristics or at least developed similar ones. While in Europe Albert was one of his favorites and from him Dr. Murphy perhaps learned how to present clinical surgery in a clear, fascinating, and logical manner. From these men and from Christian Fenger, who was a wonderful teacher of pathology, Murphy obtained a fundamental ground work in surgery which was rendered evident in all his subsequent remarkable career.

With his fertile, imaginative brain he soon began to evolve useful digressions from the conventional surgery of the day. So logical was his work that many of his earlier works remain to-day as classical monuments of his scientific ability. No specialty or special line of endeavor limited his usefulness

His early study of the intestines, with the discovery of the "button" which bears his name was soon followed by equally great advances in surgery of the appendix, gall, bladder, stomach, kidney, lungs, nervous system, bones and joints. He possessed not only technical skill to a fine degree but a comprehensive understanding of pathologic processes rarely found in surgeons. This made his teachings and writings well worth while.

There is doubtless no surgeon better known or held in higher esteem than was Dr. John B. Murphy, nor was there one whose writings are more carefully and pofitably read.

He seemed to radiate with knowledge and to impart it with contagious enthusiasm.

His judicial mind seemed to enable to judge well the relative value of things as well as men.

"He was the human man, developing, through many years of self-discipline, indefatigable work, untold sacrifice, and desire for the highest service, into the superman—the high priest of the most exacting profession."

